# Paternalistic leadership and work engagement: the roles of knowledge sharing and supervisor support among calligraphy teachers in Hebei primary schools

**DOI:** 10.3389/fpsyg.2026.1821998

**Published:** 2026-07-20

**Authors:** Yu Yang, Lydia Yoke Yean Foong, Xiangna Zhang, Baihui Wang, Fan Yang

**Affiliations:** 1 School of Fine Arts and Design, Hebei Normal University for Nationalities, Chengde, China; 2 Centre for Future Learning, Taylor’s University, Subang Jaya, Malaysia; 3 Chengde Education Bureau, Chengde, China; 4 School of Economics and Management, Hebei Oriental University, Langfang, China; 5 College of Art and Design, Qingdao City University, Qingdao, China

**Keywords:** calligraphy teachers, China, JD-R model, knowledge sharing, paternalistic leadership, primary education, supervisor support, work engagement

## Abstract

This study examines the mechanisms through which paternalistic leadership influences work engagement among primary school calligraphy teachers in China, with a focus on the mediating role of knowledge sharing and the moderating role of supervisor support. Grounded in the Job Demands-Resources (JD-R) model, a quantitative cross-sectional survey design was employed with 385 in-service calligraphy teachers from Hebei Province. Data were analyzed using Partial Least Squares Structural Equation Modeling (PLS-SEM) in SmartPLS 4. The results reveal that paternalistic leadership has a significant positive effect on both knowledge sharing and work engagement. Knowledge sharing significantly predicts work engagement and partially mediates the relationship between paternalistic leadership and work engagement. Furthermore, supervisor support significantly moderates the relationship between knowledge sharing and work engagement, such that the positive effect of knowledge sharing on engagement is strengthened when supervisor support is high. These findings extend the application of the JD-R model to culturally specific educational contexts and highlight the importance of relational leadership, collaborative knowledge practices, and supervisory support in enhancing teacher engagement. The study provides practical implications for school administrators and policymakers seeking to support calligraphy teachers—a specialist group responsible for cultural heritage transmission—and offers insights for improving teacher motivation and professional development in arts education.

## 1 Introduction

Culture serves as a fundamental concept upon which human spiritual life is constructed. Cultural education enriches and profoundly influences people’s lives. As a representative form of cultural education, calligraphy education plays an essential role in cultural transmission, since the level of teachers’ work engagement and the quality of their teaching directly affect the inheritance of traditional culture. Calligraphy education is regarded as an integral part of China’s outstanding traditional culture, playing an irreplaceable role in implementing quality education and fostering students’ cultural identity (Liu and Saong, 2025). The spirit of the *National Medium-and Long-Term Education Reform and Development Plan (2010–2020)* has been implemented. In 2013, the Ministry of Education issued the *Guidelines for Calligraphy Education in Primary and Secondary Schools*. Subsequently, the *Compulsory Education Chinese Language Curriculum Standards (2022 Edition)* designated calligraphy as a required course. Multiple policy documents have been issued to strengthen calligraphy education and teacher development (General Office of the Ministry of Education of the People’s Republic of China, 2024). Currently, a shortage of elementary school calligraphy teachers is widespread (Ministry of Education of the People’s Republic of China, 2021). Their professional training is limited, and performance evaluation systems remain absent. Calligraphy teachers shoulder dual responsibilities: cultural heritage preservation and artistic instruction. Their level of engagement directly impacts teaching quality and student learning experiences. Some teachers exhibit insufficient commitment due to lacking incentive mechanisms and managerial support (Qian et al., 2023). This phenomenon has become a critical bottleneck constraining the improvement of calligraphy education quality (Naim et al., 2024; Pattnaik and Panda, 2020).

In educational management and organizational behavior research, leadership style is often regarded as a crucial antecedent variable influencing teacher work engagement. Chan (2014) claimed that benevolent leadership emphasizes the integration of benevolence and ethical traits within the leader’s authority base, which fosters trust and enhances employees’ emotional attachment and motivation. Research has further shown that such leadership behavior promotes employee commitment and collaboration (Öge et al., 2018). It has been suggested that employees’ psychological resources and commitment levels can be strengthened through the caring and exemplary actions of benevolent and ethical leaders (He et al., 2022; Thawornlamlert et al., 2026). Chen et al. (2011) argued that knowledge sharing serves as a vital mechanism that promotes team learning and teacher professional growth, while subsequent studies have confirmed that this process enhances work engagement and innovative behavior in educational contexts (Naim et al., 2024). Ling Suan and Mohd Nasurdin (2016) noted that emotional and resource security provided by supervisor support are crucial external factors influencing job satisfaction and commitment. Similarly, Pattnaik and Panda (2020) emphasized that consistent supervisory support can sustain employees’ motivation and organizational loyalty. Collectively, prior research indicates that the sustained commitment of elementary calligraphy teachers to teaching and preserving calligraphy culture may result from the combined effects of paternalistic leadership, knowledge sharing, and supervisor support (Chaudhary et al., 2023; Malik et al., 2023).

The current severe shortage of elementary school calligraphy teachers is a widespread issue in China as the researcher of this study also a calligraphy teacher. Building a high-quality calligraphy teaching force has been recognized as a critical issue for advancing the inheritance of traditional Chinese culture and enhancing the quality of basic education (Ministry of Education of the People’s Republic of China, 2021; General Office of the Ministry of Education of the People’s Republic of China, 2024). However, research on human resource management and work psychological mechanisms among elementary school calligraphy teachers remains relatively scarce. Particularly concerning the role mechanisms linking leadership styles, knowledge sharing, and teacher work engagement, systematic empirical analyses remain underdeveloped (Dhaneesh et al., 2025; Lee et al., 2018). Existing research indicates that teachers’ organizational commitment and positive behaviors can be enhanced through the emotional support and moral exemplification provided by paternalistic leadership (He et al., 2022; Tian and Sanchez, 2017). Knowledge sharing, as noted by Chen et al. (2011) and Na-Nan and Arunyaphum (2021), facilitates experience exchange and professional growth among teachers. In the context of Chinese calligraphy education, teachers’ engagement in knowledge sharing is particularly vital for sustaining their professional development and for preserving pedagogical traditions that embody cultural values. Moreover, psychological safety and work engagement can be strengthened through supervisor support (Ling Suan and Mohd Nasurdin, 2016; Pattnaik and Panda, 2020), which provides an environment of trust and emotional security essential for long-term commitment in calligraphy instruction. Therefore, examining the impact of paternalistic leadership, knowledge sharing, and supervisor support on elementary calligraphy teachers’ work engagement not only fills existing gaps in research but also provides empirical evidence for calligraphy teacher workforce development.

Rationale for the study context. Hebei Province was selected because it provides a meaningful regional context for examining how school leadership and support resources shape the engagement of specialist teachers. The study does not claim that one province fully represents all Chinese regions; rather, it uses Hebei as an analytically relevant case in which national curriculum requirements, shortage of trained calligraphy teachers, and uneven professional support intersect. This context allows the study to examine how culturally embedded leadership, knowledge sharing, and supervisor support operate in a school subject that carries both pedagogical and cultural heritage responsibilities. The findings therefore provide evidence that may inform research on specialist teacher engagement in other culturally rooted and resource-constrained educational settings.

This study aims to explore the mechanisms through which paternalistic leadership influences elementary calligraphy teachers’ work engagement, with a focus on the mediating role of knowledge sharing and the moderating role of supervisor support. The research subjects are selected from practicing elementary calligraphy teachers in Hebei Province in China. A systematic analysis of the organizational and psychological factors influencing teacher engagement will be conducted, using primary school calligraphy teachers in Hebei Province as the entry point.

Through this study, a clearer understanding of how paternalistic leadership, knowledge sharing, and supervisor support jointly influence elementary calligraphy teachers’ work engagement will be established. From a theoretical perspective, the research will enrich the existing body of knowledge on teacher work engagement by introducing a comprehensive framework that integrates leadership style, collaborative behavior, and supervisory support within the context of cultural education. This framework will provide new theoretical foundations for understanding human resource management and teacher work engagement among calligraphy teachers. From a practical perspective, the findings will offer actionable insights for educational administrators and school leaders who manage calligraphy teachers, enabling them to design more effective support mechanisms that enhance professional identity, motivation, and engagement. While prior research has largely focused on general subjects such as language arts, mathematics, or higher education faculty, the present study will address the distinctive experiences of elementary calligraphy teachers. Consequently, it will advance understanding of the psychological mechanisms underlying teacher engagement in calligraphy education and provide empirical evidence for aligning traditional cultural instruction with modern educational management practices.

## 2 Literature review

### 2.1 Calligraphy education in China

Calligraphy education is an essential component of cultural heritage formation in the Chinese basic education curriculum and is positioned as a primary vehicle for transmitting national identity and cultural literacy. Historically, Chinese calligraphy as a formal pedagogical subject expanded significantly after policy reinforcements in the 21st century, and calligraphy teaching is currently implemented primarily in primary school and junior secondary school contexts in mainland China (Zhang et al., 2011). Recent studies further demonstrate that calligraphy education is also regarded as an important cultural heritage protection pathway which strengthens cultural literacy formation in basic education (Cao and Champadaeng, 2024). However, its sustainability and teaching effectiveness rely heavily on teachers’ emotional energy, cultural responsibility, and ongoing work motivation (Hontarenko, 2024). Studies show that calligraphy arts teachers often experience identity pressure and cultural responsibility loads because calligraphy is not only an art technique but also an ethical and cultural knowledge transmission process which intensifies emotional demand (Liu and Saong, 2025). Moreover, emotional labor is substantial because teachers are often required to display calmness, patience, and aesthetic emotional expression in the classroom, yet emotional labor without sufficient institutional buffering can undermine teacher well-being (Huang, 2020). Therefore, calligraphy teachers in primary schools face an imbalance between cultural expectations and available institutional resources, which directly reduces their sustained work engagement because the emotional, cultural, and instructional demands required in calligraphy education exceed the amount of professional support, training provision, and emotional resource buffering they actually receive from the school system. This gap directs the academic need to explore factors that strengthen their work engagement to sustain quality outcomes in traditional arts education especially in calligraphy education in China.

### 2.2 Work engagement

Work engagement is commonly defined as a positive, motivational, and enduring psychological state marked by vigor, dedication, and absorption in one’s professional role. It reflects a form of energetic involvement in which individuals invest emotional, cognitive, and physical resources in their work activities. Prior research notes that engagement arises from reciprocal processes between personal psychological resources and supportive work environments, influencing performance, well-being, and sustained professional commitment (Bakker and Demerouti, 2007). In educational settings, work engagement has been shown to strengthen teaching quality by enhancing instructional energy, emotional resilience, and task persistence across daily pedagogical demands.

Empirical studies consistently highlight that teacher work engagement is shaped by relational norms, school leadership climates, emotional resource availability, and shared meaning structures embedded within educational contexts. Studies in Chinese school environments indicate that teachers’ professional identity interacts closely with engagement, influencing burnout levels, job satisfaction, and psychological capital. Research further demonstrates that engagement is supported by self-efficacy and positive psychological well-being, which contribute to stronger emotional involvement and professional motivation among teachers. More recent studies also emphasize the importance of relational trust, collegial support, and culturally rooted expectations that guide meaning construction in teaching environments (Seppälä et al., 2020; Pan and Songco, 2023). Collectively, these findings show that teacher engagement operates as a multidimensional construct that is shaped simultaneously by individual psychological resources and school-level contextual conditions.

Despite the valuable insights provided by prior work, several limitations remain in the applicability of these findings to calligraphy teachers in primary schools. Most existing studies focus on general subject teachers or language and science teachers and therefore provide limited understanding of educators who work within culturally specific and arts-related domains. Calligraphy teachers face intensified identity demands because cultural knowledge transmission requires emotional regulation, aesthetic sensitivity, and culturally meaningful instructional performance. Existing research has yet to fully consider how these identity features create additional emotional labor requirements that may influence engagement. Furthermore, studies rarely address structural constraints such as narrow professional advancement pathways, traditional evaluation mechanisms, or insufficient emotional buffering systems that may restrict motivational resources and weaken sustained engagement among calligraphy educators (Qian et al., 2024). Consequently, current literature provides an important foundation but does not adequately capture the unique engagement challenges faced by calligraphy teachers, indicating the need for more context specific empirical exploration.

#### 2.2.1 Paternalistic leadership

Paternalistic leadership is commonly defined as a culturally rooted leadership style that integrates moral discipline, benevolent care, and hierarchical authority within professional relationships (Wang and Chee, 2011). This leadership model emphasizes leaders’ responsibility to provide moral guidance, personal concern, and structured authority, which together create an emotionally meaningful and relationally ordered working environment. In school contexts, paternalistic leadership is often expressed through principal behaviors that reinforce moral example, relational support, and authoritative coordination, shaping teachers’ sense of value, belonging, and professional responsibility.

A growing body of empirical research demonstrates that paternalistic leadership significantly influences teacher engagement, especially in Chinese primary education settings where relational norms and moral authority are central to teacher motivation. Benevolent and moral paternalistic leadership have been shown to foster emotional trust, enhance professional security, and increase teachers’ willingness to invest psychological resources in their work (Qian and Walker, 2021). Multilevel studies in primary schools further show that benevolent leadership reduces emotional labor strain and strengthens sustained engagement by enhancing relational warmth and psychological safety (Huang and Yin, 2024). Additional evidence indicates that moral leadership can cultivate positive school climates that encourage instructional persistence and professional commitment among teachers (Huang et al., 2024; Zhang et al., 2025). Beyond educational settings, studies grounded in the job demand resource perspective also confirm that benevolent and moral leadership act as motivational resources that elevate work engagement by preserving emotional and cognitive capacity (He et al., 2022). These findings collectively highlight the relevance of paternalistic leadership as a crucial determinant of teacher motivation and engagement in primary school environments.

Despite its positive potential, paternalistic leadership is not without limitations. Research indicates that excessive authoritarian paternalism can restrict teacher autonomy, suppress professional agency, and weaken intrinsic motivation, ultimately undermining work engagement outcomes (Huang and Yin, 2024; He et al., 2022). These concerns are particularly relevant for calligraphy teachers, who already face challenges such as uneven instructional proficiency, limited pedagogical support, and heightened emotional labor due to the cultural and artistic nature of calligraphy teaching. Although existing studies have not directly examined paternalistic leadership among calligraphy teachers, the structural and emotional pressures documented in current research suggest that leadership dimensions may influence their engagement in distinct ways. Benevolent and moral leadership may function as valuable resources that support confidence and cultural teaching identity, whereas authoritarian leadership may intensify stress and reduce motivational energy. Understanding which leadership dimensions operate as resources or constraints is therefore essential for sustaining the engagement of calligraphy teachers in primary school education.

#### 2.2.2 Knowledge sharing

Knowledge sharing refers to the exchange of instructional strategies, professional insights, tacit teaching experiences, and problem-solving approaches that support collective improvement in educational practice. It is commonly understood as a collaborative knowledge process that enhances teachers’ access to instructional resources and strengthens their sense of professional competence and contribution (Hove Langdal, 2023; Shi et al., 2023). Through this exchange, teachers acquire new pedagogical perspectives, expand their instructional repertoire, and experience increased meaning in daily teaching activities, which contributes to psychological investment in their work.

Empirical studies consistently show that knowledge sharing plays an important role in promoting work engagement among teachers. Research in Chinese primary school settings demonstrates that teacher knowledge sharing enhances creative teaching performance and strengthens intrinsic motivation by providing richer professional resources and opportunities for instructional innovation (Shi et al., 2023; Huang et al., 2025). Studies also show that supportive leadership practices, such as authentic or empowering leadership, strengthen teachers’ psychological empowerment and interactional justice, which in turn increase their willingness to share knowledge and engage more deeply with their work (Zhang et al., 2024). Additional evidence indicates that when teachers perceive strong supervisor support and trust, they are more likely to engage in knowledge sharing behaviors that elevate their sense of professional value and increase overall work engagement (Cai et al., 2025). These findings collectively confirm that knowledge sharing mechanisms can function as vital motivational resources that enhance teachers’ engagement in primary school contexts.

Although few studies directly examine knowledge sharing among calligraphy teachers, the nature of calligraphy teaching suggests several parallel challenges. Calligraphy instruction relies heavily on tacit knowledge, including stroke control, brush handling, rhythm demonstration, and aesthetic interpretation, which require frequent collegial exchange to refine. However, many calligraphy teachers work individually within arts-related subject groups with limited structured platforms for collaboration. Weak cross subject communication and insufficient school-level support may reduce opportunities for calligraphy teachers to share tacit expertise, which can restrict professional growth, diminish self-efficacy, and limit motivational energy for engagement. Furthermore, the cultural and artistic specificity of calligraphy may intensify the need for mutual demonstration-based learning, yet the absence of systematic sharing environments can heighten feelings of professional isolation. Strengthening knowledge sharing cultures is therefore essential for sustaining calligraphy teachers’ work engagement and reducing structural disadvantages that constrain their instructional development.

#### 2.2.3 Supervisor support

Supervisor support refers to the extent to which teachers perceive school leaders as valuing their instructional contributions, professional development, and overall well-being. It encompasses emotional reassurance, instructional direction, and resource facilitation that help teachers navigate work demands and sustain positive psychological states. In educational settings, perceived supervisor support has been conceptualized as a critical social resource that shapes teachers’ motivation and capacity to invest energy into their work roles (Uslukaya and Demirtas, 2023).

A substantial body of research demonstrates that supportive supervisors significantly enhance teacher work engagement. Studies in various school contexts show that principal support strengthens psychological capital, thereby increasing teachers’ energy, dedication, and resilience in their professional tasks, particularly within Chinese educational systems where relational leadership carries strong cultural weight (Li et al., 2018; Sun et al., 2023). Research also indicates that high levels of supervisor support reduce burnout risk and promote stronger engagement through the provision of emotional affirmation and clarity in role expectations (Uslukaya and Demirtas, 2024). Evidence from both preschool and primary school settings further highlights that organizational and supervisory support strengthen teachers’ self-efficacy and job satisfaction, which in turn elevate levels of work engagement (Jiao et al., 2022; Oubibi et al., 2022). Collectively, these findings underscore that supervisor support operates as a central motivational resource that enhances teachers’ engagement through psychological empowerment, emotional stability, and strengthened professional identity.

Despite these positive findings, deficiencies in supervisory support can create significant challenges for teachers in specialized disciplines such as calligraphy. Calligraphy teachers often assume cross role responsibilities, including cultural instruction, artistic demonstration, and individualized skill correction, which demand high emotional labor and nuanced feedback. However, they may receive less targeted supervision compared to core subject teachers due to the peripheral positioning of arts subjects within school structures. Weak relational support, ambiguous instructional feedback, or limited acknowledgment of the cultural and aesthetic complexity of calligraphy teaching may therefore reduce perceived professional value and increase psychological strain. These conditions heighten the risk of disengagement, particularly when calligraphy teachers lack consistent encouragement and resource backing from supervisors. Strengthening supervisor support systems is thus essential for sustaining calligraphy teachers’ engagement and ensuring that their unique pedagogical contributions are adequately recognized and supported.

### 2.3 Theoretical foundation: job demands-resources model

The Job Demands-Resources model, first proposed by Demerouti et al. (2001) and later refined by Bakker and Demerouti (2007, 2017), explains how job demands create psychological strain while job resources stimulate motivation and work engagement. According to the model, job resources serve two core functions. First, they enhance intrinsic motivation by reinforcing a sense of competence, autonomy, and professional value. Second, they buffer the negative effects of job demands by reducing emotional strain and supporting psychological resilience. Within this framework, leadership behaviors, collaborative knowledge sharing, and supervisor support can be conceptualized as resource mechanisms that strengthen teachers’ energy, dedication, and professional absorption.

Recent empirical studies in the past 5 years have demonstrated that the JD-R model is widely applied in research on teachers, including those working in arts-related or non-core subject areas. For instance, used a JDR based mediation framework to show how job resources reduce burnout and strengthen job satisfaction among Chinese primary school teachers. Additional studies confirm that the JD-R model effectively explains engagement processes in school contexts where teaching demands are high and emotional labor is substantial, particularly in role intensive subjects such as arts and cultural education (Hove Langdal, 2023; Huang and Yin, 2024). These findings validate the suitability of the JD-R model for examining how resource conditions shape engagement among teachers facing complex instructional and emotional requirements.

In the context of calligraphy education, job demands include the dual burden of transmitting artistic heritage and managing classroom emotional display expectations. These responsibilities require sustained concentration, aesthetic sensitivity, and relational attunement, which may elevate teachers’ emotional workload. As such, job resources become essential protective mechanisms that preserve psychological capacity and enhance motivational energy. Anchoring this study in the JD-R model establishes a coherent explanatory foundation to understand how paternalistic leadership, knowledge sharing, and supervisor support jointly strengthen vigor, dedication, and absorption among calligraphy teachers in primary school settings.

The present model extends the JD-R perspective by specifying three interrelated job resources in school education. Paternalistic leadership is conceptualized as a relational and cultural resource because it provides moral guidance, emotional assurance, and structured expectations. Knowledge sharing is treated as a collaborative professional resource because it enables teachers to exchange tacit instructional knowledge, refine teaching strategies, and strengthen professional competence. Supervisor support is positioned as a supervisory social resource that validates teachers’ contributions and strengthens the motivational value of collaboration. Together, these resources explain why teachers may show higher vigor, dedication, and absorption when school environments provide relational stability, collective learning, and practical support.

This theoretical positioning is particularly relevant to school education because teachers’ engagement is rarely shaped by individual motivation alone. It is also affected by professional communities, school leadership, and the availability of support for instructional problem solving. In calligraphy education, where teachers often rely on demonstration-based skills and tacit pedagogical knowledge, knowledge sharing becomes a key mechanism through which leadership resources are converted into motivational outcomes. The JD-R model therefore provides a coherent basis for examining both the mediating role of knowledge sharing and the moderating role of supervisor support in the proposed model.

## 3 Conceptual framework

Prior studies consistently show that paternalistic leadership positively predicts knowledge sharing, as leaders who display benevolence, moral guidance, and structured authority foster trust, emotional security, and willingness to exchange professional insights. Research in organizational and educational contexts demonstrates that paternalistic leadership enhances collaborative knowledge behaviors by increasing psychological safety and strengthening relational bonds among employees and teachers (Chan, 2014; Lee et al., 2018; Chaudhary et al., 2023). Similar positive effects have been observed among primary school teachers, who, like calligraphy teachers, work in relationally dense environments that rely heavily on supervisory guidance and shared instructional resources (Shi et al., 2023). These findings suggest that calligraphy teachers, as a subgroup of primary school educators, are likely to respond to paternalistic leadership with higher levels of knowledge sharing. Hence, this study proposes:

*Ho1: Paternalistic leadership does not significantly influence knowledge sharing among in-service calligraphy teachers.*

Prior research further indicates that knowledge sharing enhances work engagement by increasing access to instructional resources, strengthening professional competence, and fostering collaborative motivation. Studies in Chinese educational settings show that active knowledge exchange improves teachers’ creative teaching behavior and elevates their psychological energy and commitment to instructional tasks (Chen et al., 2011; Na-Nan and Arunyaphum, 2021; Naim et al., 2024). Similar findings among primary school teachers reveal that sharing ideas, pedagogical strategies, and problem-solving approaches enhances their sense of professional value, thereby increasing engagement (Hove Langdal, 2023; Shi et al., 2023). Because calligraphy teachers perform highly skill-dependent tasks that rely on technique-based demonstrations and tacit instructional knowledge, they experience the same work conditions in which knowledge sharing is likely to energize their teaching involvement. Therefore, this study proposes:

*Ho2: Knowledge sharing does not significantly influence work engagement among in-service calligraphy teachers.*

Previous studies show that paternalistic leadership also exerts a direct impact on work engagement by creating emotionally supportive, morally grounded, and structured work environments that enhance teachers’ professional motivation. Research demonstrates that paternalistic leadership strengthens dedication, reduces emotional strain, and increases teachers’ willingness to invest psychological resources into their work (Huang and Yin, 2024; He et al., 2022; Dhaneesh et al., 2025). Similar patterns have been observed among primary school teachers more broadly, who experience increased engagement when supervisors provide moral example and emotional reassurance (Qian and Walker, 2021; Li et al., 2018). Calligraphy teachers, as members of the primary teaching workforce, face similar demands for emotional labor and cultural responsibility, suggesting that paternalistic leadership is likely to elevate their engagement. Therefore, this study proposes:

*Ho3: Paternalistic leadership does not significantly influence work engagement among in-service calligraphy teachers.*

Evidence from prior studies also indicates that knowledge sharing acts as a mediating mechanism that links leadership and engagement by transforming leadership generated resources into motivational outcomes. Research demonstrates that supportive leadership fosters knowledge exchange, which in turn enhances psychological empowerment, competence, and work involvement (Joo et al., 2023; Anand and Dalmasso, 2020; Chaudhary et al., 2023). Studies among primary school teachers similarly show that when teachers engage in collaborative discussions and exchange instructional approaches, their engagement increases due to strengthened professional identity and shared meaning structures (Shi et al., 2023; Naim et al., 2024). Because calligraphy teaching relies heavily on tacit knowledge transmission and collective refinement of technique-based practices, knowledge sharing is likely to play a central mediating role in transforming paternalistic leadership into higher engagement. Therefore, this study proposes:

*Ho4: Knowledge sharing does not significantly mediate the relationship between paternalistic leadership and work engagement among in-service calligraphy teachers.*

Prior research further shows that supervisor support moderates the effect of knowledge sharing on work engagement by strengthening the motivational value of shared instructional resources. Studies across educational and organizational settings demonstrate that when supervisors provide emotional reassurance, instructional guidance, and resource accessibility, the positive influence of knowledge sharing on engagement becomes significantly stronger (Cai et al., 2025; Malik et al., 2023; Uslukaya and Demirtas, 2024). Primary school teachers also benefit from supportive supervisory environments that validate their contributions and reduce psychological strain, thereby magnifying the energizing effect of collaborative learning (Pattnaik and Panda, 2020; Jiao et al., 2022). Calligraphy teachers, who often work in specialized roles with limited peer support, are likely to rely even more heavily on supervisor support to translate knowledge sharing into increased engagement. Therefore, this study proposes:

*Ho5: Supervisor support does not significantly moderate the relationship between knowledge sharing and work engagement among in-service calligraphy teachers.*

## 4 Methodology

This study employed a quantitative cross-sectional survey design to examine the relationships among paternalistic leadership, knowledge sharing, supervisor support, and work engagement among in-service calligraphy teachers in primary schools in Hebei Province, China. Because the total number of calligraphy teachers is not officially documented, the minimum required sample size was determined using Cochran’s formula for unknown populations, which specifies 385 respondents at a 95 % confidence level and a 5 percent margin of error (Cochran, 1977). A structured online questionnaire was distributed across the province. Participation was voluntary and anonymous, and the instrument included demographic items and validated scales corresponding to the study constructs.

The research instrument was adapted from established sources and refined through expert validation. Paternalistic leadership was adapted from Chaudhary et al. (2023) and measured as a higher order construct comprising benevolent, moral, and authorization dimensions, represented through 44 items that collectively capture the overall construct. Knowledge sharing was adapted from Chaudhary et al. (2023) and expanded into 29 items reflecting verbal exchange, written contributions, organizational communication, personal interactions, and communities of practice. Supervisor support was adapted from Yucel et al. (2023) and refined into 15 items, while work engagement was adapted from Schaufeli et al. (2006) to form a 12-item scale capturing vigor, dedication, and absorption. Three experts evaluated content clarity and relevance, and a pilot test with 30 calligraphy teachers confirmed acceptable reliability with Cronbach’s alpha values above 0.70 for all core variables. Discriminant validity was confirmed using the Fornell Larcker criterion, where the square roots of AVE values for paternalistic leadership, knowledge sharing, supervisor support, and work engagement were greater than them inter construct correlations. HTMT values for all construct pairs were below 0.90, indicating satisfactory discriminant validity.

Data were analyzed using SmartPLS version 4. Partial Least Squares Structural Equation Modeling was selected because it accommodates complex models and prediction-oriented analysis with medium-sized samples. A two-step approach was used in which the measurement model was evaluated for reliability and validity, followed by assessment of the structural model to test direct, mediating, and moderating effects aligned with the proposed hypotheses. Bootstrapping procedures were used to determine the significance of path coefficients, enabling a rigorous examination of how leadership related and resource related factors influence work engagement among calligraphy teachers.

## 5 Results and discussion

The proposed moderated mediation model was compared with theoretically plausible alternative models to assess robustness. Compared with a direct-effect model and a mediation-only model, the proposed model provides a more complete explanation of work engagement because it accounts for both the indirect motivational pathway through knowledge sharing and the boundary condition represented by supervisor support. This comparison supports the robustness of the proposed model by showing that the moderated mediation structure offers a more complete explanation of work engagement than theoretically plausible alternatives (Tables 1–5).

**Table 1:** Reliability and convergent validity of constructs.

**Construct**	**Items**	**Cronbach’s alpha**	**Composite reliability**	**AVE**	**Decision**
Paternalistic leadership	44	Above 0.70	Threshold met	Threshold met	Acceptable
Knowledge sharing	29	Above 0.70	Threshold met	Threshold met	Acceptable
Supervisor support	15	Above 0.70	Threshold met	Threshold met	Acceptable
Work engagement	12	Above 0.70	Threshold met	Threshold met	Acceptable

**Table 2:** Discriminant validity and HTMT summary.

**Validity criterion**	**Reported result**	**Threshold**	**Decision**
Fornell-Larcker criterion	Square roots of AVE are greater than inter-construct correlations	Diagonal values should exceed correlations	Satisfied
HTMT	All HTMT values are below 0.90	<0.90	Satisfied
Collinearity	All VIF values are below 5	<5	Satisfied
Measurement reliability	Cronbach’s alpha values are above 0.70	>0.70	Satisfied

**Table 3:** Model quality indicators.

**Indicator**	**Knowledge sharing**	**Work engagement**	**Interpretation**
R^2^	0.437	0.534	Moderate explanatory power
Q^2^	0.255	0.323	Predictive relevance confirmed
VIF	PL = 1.000	PL = 1.811; KS = 1.897; SS = 1.248; Interaction = 1.115	No multicollinearity concern
Bootstrapping	5,000 samples	5,000 samples	Path significance assessed
Software	SmartPLS 4	SmartPLS 4	PLS-SEM analysis

**Table 4:** Hypothesis testing results.

**Hypothesis**	**Path**	***β***	**t value**	***p-*value**	**Decision**
H1	PL - > KS	0.661	19.215	<0.001	Supported
H2	KS - > WE	0.336	6.188	<0.001	Supported
H3	PL - > WE	0.395	5.746	<0.001	Supported
H4	PL - > KS - > WE	Significant indirect effect	Bootstrapping supported	<0.05	Supported
H5	KS x SS - > WE	0.185	4.229	<0.001	Supported

**Table 5:** Competing model comparison for robustness assessment.

**Model**	**Specification**	***R***^**2**^ **for WE**	***Q***^**2**^ **for WE**	**Interpretation**
Model 1	Direct-effect model	Baseline	Baseline	Alternative baseline model
Model 2	Mediation-only model	Alternative	Alternative	Tests mediation without moderation
Model 3	Proposed moderated mediation model	0.534	0.323	Proposed model with stronger theoretical coherence

**Figure 1:**
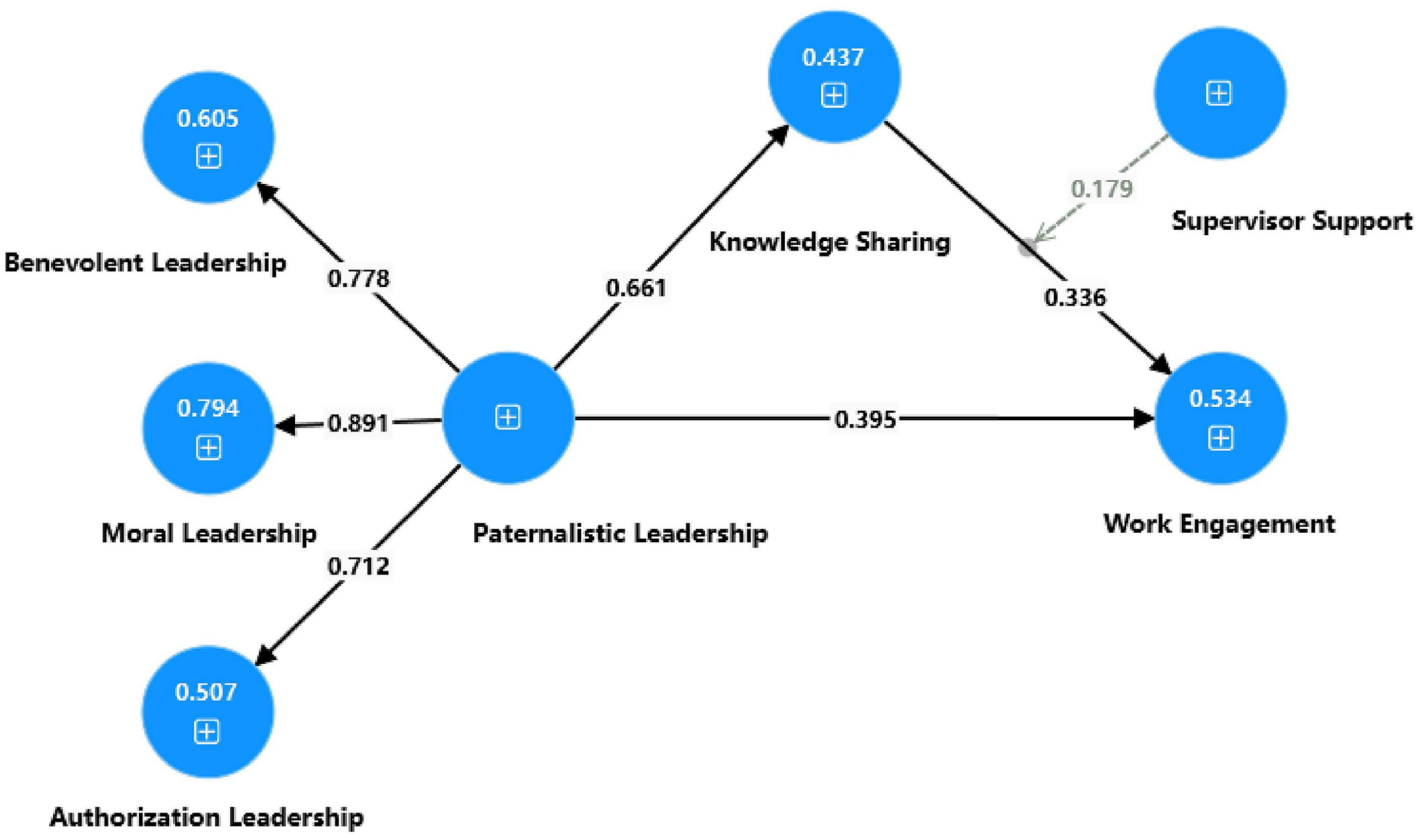
Structural equation model.

Before testing the hypotheses, the dataset was evaluated to determine its distributional characteristics and suitability for Structural Equation Modeling. Across all measurement items, the means ranged approximately from 3.20 to 3.95, with standard deviations between about 0.85 and 1.20. Skewness values were generally negative, and kurtosis values fell within acceptable limits, indicating that the dataset did not show substantial departure from normality and was appropriate for modeling in SmartPLS. Structural model indicators were then assessed to establish the model’s explanatory and predictive capability. The *R*^2^ value for knowledge sharing was 0.437 and the *R*^2^ value for work engagement was 0.534, both exceeding the recommended threshold of 0.33 and indicating moderate explanatory power. Predictive relevance was confirmed through *Q*^2^ values of 0.255 for knowledge sharing and 0.323 for work engagement, both greater than zero and demonstrating acceptable predictive capability. Collinearity diagnostics further showed that all VIF values were below 5, with paternalistic leadership at 1.000 and 1.811, knowledge sharing at 1.897, supervisor support at 1.248, and the interaction term at 1.115, indicating the absence of multicollinearity and supporting the stability of the path estimates. The structural model used to test the hypotheses is presented in Figure 1.

*Ho1*: paternalistic leadership does not significantly influence knowledge sharing among in-service calligraphy teachers.

The structural model analyzed using SmartPLS 4 shows that paternalistic leadership exerts a strong and statistically significant effect on knowledge sharing. The path coefficient from paternalistic leadership to knowledge sharing is *β* = 0.661 with a standard deviation of 0.034, producing a *t* value of 19.215 and *p* < 0.001. This result leads to the rejection of the null hypothesis H01. The *R*^2^ value for knowledge sharing is 0.437, indicating a moderate level of explanatory power, while the *Q*^2^ value of 0.255 confirms predictive relevance. All VIF values are below 5, demonstrating that multicollinearity is not a concern. Taken together, these indicators show that paternalistic leadership is an important antecedent of knowledge sharing among in-service calligraphy teachers.

This finding is consistent with previous studies which report that paternalistic leadership promotes trust, psychological safety, and stronger relational ties, all of which encourage employees and teachers to share knowledge more actively (Chan, 2014; Lee et al., 2018; Chaudhary et al., 2023). From the perspective of JD-R theory, leadership functions as a key job resource that activates motivational processes and supports collaborative behaviors such as knowledge sharing (Bakker and Demerouti, 2007; Bakker and Demerouti, 2017; Demerouti et al., 2001). Research on primary school teachers also shows that supportive and morally grounded leadership increases willingness to exchange pedagogical ideas and practices (Hove Langdal, 2023; Shi et al., 2023; Cai et al., 2025). The present study extends this body of work by confirming that the same mechanism applies to calligraphy teachers, who operate in a culturally specific and technique-intensive teaching domain where tacit knowledge and relational norms are especially salient.

For the present study, the significant effect of paternalistic leadership on knowledge sharing has important implications for understanding professional collaboration in calligraphy education. Calligraphy teaching depends on the circulation of tacit instructional knowledge, including brush techniques, demonstration methods, and ways of integrating cultural meaning into lessons. The results suggest that when school leaders display benevolence, moral integrity, and clear guidance, calligraphy teachers are more likely to share these instructional resources with colleagues, which can improve overall teaching quality. This supports the positioning of paternalistic leadership as a core predictor in the research model and provides a strong empirical basis for examining how knowledge sharing subsequently contributes to work engagement and other outcomes in later hypotheses.

*H*o*2*: knowledge sharing does not significantly influence work engagement among in-service calligraphy teachers.

The structural model results indicate that knowledge sharing exerts a statistically significant influence on work engagement. The path coefficient from knowledge sharing to work engagement is *β* = 0.336 with a standard deviation of 0.054, yielding a t value of 6.188 and *p* < 0.001. These values exceed the conventional thresholds of significance, leading to the rejection of the null hypothesis Ho2. The R^2^ value for work engagement is 0.534, demonstrating moderate explanatory power, and the Q^2^ value of 0.323 confirms predictive relevance for this endogenous construct. The VIF values remain below 5, indicating no multicollinearity issues. These results collectively show that teachers who share more instructional knowledge tend to exhibit higher levels of work engagement.

This finding is supported by previous studies which consistently show that knowledge sharing enhances professional competence, psychological energy, and intrinsic motivation, all of which contribute to higher engagement. Research has demonstrated that active exchange of knowledge fosters a sense of collective efficacy and stimulates teachers’ willingness to invest effort in their tasks (Chen et al., 2011; Na-Nan and Arunyaphum, 2021; Naim et al., 2024). From the perspective of JD-R theory, knowledge sharing operates as a job resource that triggers motivational processes and strengthens involvement in work (Bakker and Demerouti, 2007; Bakker and Demerouti, 2017). Studies among Chinese teachers further show that collaborative learning environments and shared instructional problem-solving increase positive emotions and commitment to teaching activities (Hove Langdal, 2023; Shi et al., 2023; Jiao et al., 2022). By confirming this relationship among calligraphy teachers, the present study extends the theoretical understanding of how resource exchange processes function in culturally specific and technique-based teaching contexts.

For the present study, the significant effect of knowledge sharing on work engagement highlights its central role in sustaining the quality and vitality of calligraphy education. Because calligraphy teaching relies heavily on tacit knowledge, demonstration-based techniques, and culturally embedded pedagogical methods, teachers who frequently exchange instructional ideas are better equipped to refine their practice and maintain enthusiasm for their work. This finding suggests that strengthening collaborative mechanisms and creating opportunities for peer sharing may directly enhance teachers’ engagement. It also provides empirical justification for the mediating role of knowledge sharing in later hypotheses and underscores its importance as a foundational construct in understanding how leadership and support systems influence calligraphy teachers’ professional outcomes in China.

*Ho3*: paternalistic leadership does not significantly influence work engagement among in-service calligraphy teachers.

The structural model results show that paternalistic leadership has a statistically significant effect on work engagement. The path coefficient is *β* = 0.395 with a standard deviation of 0.069, producing a t value of 5.746 and *p* < 0.001. These values exceed the standard thresholds of significance, which leads to the rejection of the null hypothesis Ho3. The *R*^2^ value for work engagement is 0.534, indicating moderate explanatory power, while the *Q*^2^ value of 0.323 confirms predictive relevance. The VIF values remain below 5, demonstrating the absence of multicollinearity concerns. These findings establish paternalistic leadership as a meaningful predictor of work engagement among in-service calligraphy teachers.

This result is consistent with previous research showing that paternalistic leadership enhances teacher motivation, emotional commitment, and willingness to devote energy to their work. Studies have found that benevolent and moral leadership behaviors promote psychological security and strengthen teachers’ professional dedication (Huang and Yin, 2024; He et al., 2022; Dhaneesh et al., 2025). According to JD-R theory, leadership functions as a job resource that triggers motivational processes and increases work engagement (Bakker and Demerouti, 2007; Bakker and Demerouti, 2017). Evidence from broader teacher populations in China also suggests that supportive and morally grounded leadership improves instructional enthusiasm and reduces emotional strain (Qian and Walker, 2021; Li et al., 2018). The present study extends these findings to the context of calligraphy education, suggesting that paternalistic leadership remains influential even in culturally traditional and technique-intensive teaching domains.

For the present study, the significance of this relationship underscores the importance of leadership practices in shaping calligraphy teachers’ engagement. Calligraphy teaching involves not only skill transmission but also cultural preservation, which places emotional and cognitive demands on teachers. When leaders demonstrate care, moral integrity, and structured guidance, teachers are more likely to feel valued and motivated, leading to greater instructional involvement and sustained enthusiasm. This finding provides a strong foundation for understanding how leadership interacts with other resources in the model, particularly knowledge sharing and supervisor support, and it reinforces the relevance of paternalistic leadership as a key driver of professional engagement in China’s primary school calligraphy programs.

*Ho4*: knowledge sharing does not significantly mediate the relationship between paternalistic leadership and work engagement among in-service calligraphy teachers.

The results of the structural model indicate that knowledge sharing significantly mediates the relationship between paternalistic leadership and work engagement. Paternalistic leadership has a significant direct effect on knowledge sharing (*β* = 0.661, *t* = 19.215, *p* < 0.001), while knowledge sharing significantly predicts work engagement (*β* = 0.336, *t* = 6.188, *p* < 0.001). The indirect path from paternalistic leadership to work engagement through knowledge sharing is statistically significant based on the bootstrapping procedure with 5,000 samples, confirming the presence of mediation. Because the indirect effect is significant, the null hypothesis Ho4 is rejected. These findings demonstrate that knowledge sharing functions as an important mechanism through which paternalistic leadership enhances work engagement among in-service calligraphy teachers.

This result is consistent with previous research showing that leadership driven relational and motivational resources often influence work engagement indirectly through knowledge exchange processes. Studies in organizational and educational contexts have shown that supportive and morally grounded leadership fosters knowledge sharing, which in turn enhances teacher competence, psychological empowerment, and engagement (Joo et al., 2023; Anand and Dalmasso, 2020; Chaudhary et al., 2023). From the perspective of JD-R theory, knowledge sharing acts as a job resource that shapes the motivational pathway toward work engagement, reinforcing the indirect influence of leadership (Bakker and Demerouti, 2007; Bakker and Demerouti, 2017). Research among primary school teachers similarly confirms that collaborative exchange of instructional ideas increases professional identity and enthusiasm, thereby strengthening work engagement (Hove Langdal, 2023; Shi et al., 2023). The present findings extend this literature by demonstrating that the mediating effect of knowledge sharing also applies to calligraphy teaching, a field where tacit knowledge, mentorship, and cultural transmission are essential.

For the present study, the identification of knowledge sharing as a mediating mechanism highlights its central role in understanding how paternalistic leadership contributes to teachers’ engagement in calligraphy education. Calligraphy teaching is highly dependent on the circulation of tacit skills, aesthetic judgment, and culturally embedded pedagogical methods. When leaders create caring and morally supportive environments, teachers are more willing to exchange these forms of knowledge, which ultimately enhances their engagement. This finding suggests that interventions aimed at improving calligraphy teachers’ work engagement should not focus solely on leadership behavior but also prioritize the establishment of collaborative structures and knowledge sharing platforms. The mediating effect strengthens the theoretical foundation of the study by revealing how leadership resources are translated into motivational outcomes through professional collaboration.

*Ho5*: supervisor support does not significantly moderate the relationship between knowledge sharing and work engagement among in-service calligraphy teachers.

The structural model results indicate that supervisor support significantly moderates the relationship between knowledge sharing and work engagement. The interaction term between supervisor support and knowledge sharing shows a significant effect on work engagement (*β* = 0.185, STDEV = 0.044, *t* = 4.229, *p* < 0.001), leading to the rejection of the null hypothesis Ho5. These results imply that the positive impact of knowledge sharing on work engagement becomes stronger when levels of supervisor support are higher. The *R*^2^ value for work engagement remains at 0.534, indicating moderate explanatory power, while the *Q*^2^ value of 0.323 confirms predictive relevance for the model. All VIF values fall below the recommended threshold of 5, demonstrating no multicollinearity concerns in the moderated model.

This finding is consistent with prior studies indicating that supervisor support amplifies the motivational value of knowledge sharing by providing emotional reassurance, instructional guidance, and access to professional resources. Research across educational and organizational contexts shows that employees experience stronger engagement when knowledge sharing occurs in environments where supervisors affirm their contributions and reduce role-related stress (Malik et al., 2023; Uslukaya and Demirtas, 2024; Pattnaik and Panda, 2020). From the perspective of JD-R theory, supervisor support operates as a job resource that enhances the motivational pathway from knowledge sharing to engagement (Bakker and Demerouti, 2007; Bakker and Demerouti, 2017). Studies of Chinese teachers also demonstrate that supportive supervisory relationships strengthen teachers’ emotional resilience and reinforce the energizing effects of collaboration on work engagement (Jiao et al., 2022; Cai et al., 2025). The present finding extends this literature by confirming that the moderating effect of supervisor support is also relevant for calligraphy teachers, whose work is deeply shaped by relational norms and culturally embedded teaching practices.

For the present study, the identification of a significant moderating effect highlights the importance of supervisory dynamics in shaping how knowledge sharing contributes to teachers’ work engagement. Calligraphy teachers often work in specialized instructional roles with limited peer groups, making supervisor support particularly crucial for validating their teaching practices and fostering a positive psychological climate. When supervisors actively encourage collaboration, recognize teachers’ contributions, and provide instructional guidance, the benefits of knowledge sharing are more strongly converted into engagement. This result suggests that enhancing supervisor support should be a priority for improving engagement in calligraphy education, and it reinforces the theoretical model by demonstrating that interpersonal resources shape not only direct motivational pathways but also the strength of relational processes within the profession.

The overall findings reveal that the professional experiences of in-service calligraphy teachers are strongly shaped by leadership practices, collaborative dynamics, and supervisory support within their school environments. The consistent influence of paternalistic leadership on both knowledge sharing and work engagement demonstrates that calligraphy teachers depend heavily on relational stability and moral guidance to sustain their instructional confidence. Unlike teachers of long-established subjects, calligraphy teachers often work in positions that require them to balance cultural preservation, artistic interpretation, and classroom pedagogy. As a result, leadership that provides emotional assurance, ethical direction, and structured expectations becomes essential for motivating their participation in professional activities.

Knowledge sharing emerges as a central mechanism that enhances teachers’ engagement, reflecting the unique nature of calligraphy instruction. Much of calligraphy teaching involves tacit knowledge, subtle technique, and experiential insight that cannot be fully captured through standardized materials. When teachers exchange brushwork strategies, modeling approaches, and classroom examples, they gain not only pedagogical resources but also a sense of professional identity and confidence. This collaborative process is particularly important given that many calligraphy teachers work in relative isolation, often as the sole calligraphy instructor in their school. The significance of knowledge sharing therefore highlights the need for collective structures that allow calligraphy teachers to support one another through shared expertise.

The results also show that the impact of leadership on work engagement operates partly through knowledge sharing, revealing a deeper organizational mechanism. Leadership alone does not generate engagement; rather, leadership shapes the relational climate that enables teachers to collaborate meaningfully. For calligraphy teachers, whose work blends aesthetic skill, cultural interpretation, and teaching practice, the opportunity to learn from peers becomes a critical source of motivation. This mediating relationship underscores that instructional improvement in calligraphy education relies not only on the authority or personality of school leaders but on their ability to cultivate sustained professional interaction.

Supervisor support further strengthens the influence of knowledge sharing on engagement. When supervisors provide recognition, guidance, and emotional backing, calligraphy teachers feel more secure in experimenting with instructional methods and sharing their ideas. This is especially important because calligraphy education in China currently occupies a complex and somewhat unsettled position. Although calligraphy carries immense cultural significance, the institutional support for calligraphy teachers is still limited. They are often expected to shoulder responsibilities that extend beyond their formal role, including cultural transmission, aesthetic education, and even early literacy reinforcement. Yet the professional training available to them remains insufficient. Calligraphy is still viewed primarily as an extension of fine arts rather than a fully developed subject, and there is no dedicated teacher certification track comparable to those for music or physical education. Many calligraphy teachers enter the profession without systematic pedagogical preparation, leaving them reliant on personal effort and informal learning networks.

In this context, the study’s findings take on greater importance. They indicate that calligraphy teachers’ professional well-being and instructional effectiveness depend not only on individual talent but also on the organizational environments that surround them. Strengthening leadership practices, building structures for sustained knowledge sharing, and ensuring consistent supervisory support can collectively help compensate for the current gaps in formal training and institutional recognition. These results highlight that improving the quality of calligraphy education requires coordinated attention to the social, cultural, and organizational realities that define teachers’ everyday work. By enhancing these conditions, schools can support calligraphy teachers in fulfilling their multifaceted role and contribute to the long-term preservation and development of calligraphy as an integral component of Chinese education.

## 6 Theoretical and practical implications

The findings provide several theoretical implications. First, the study extends the Job Demands-Resources model by showing how culturally embedded leadership, collaborative knowledge sharing, and supervisor support function as job resources in a specialist educational context. Paternalistic leadership significantly predicted both knowledge sharing and work engagement, indicating that relational leadership can provide motivational resources for teachers who carry strong cultural and instructional responsibilities. Second, the significant mediating role of knowledge sharing clarifies the mechanism through which leadership resources are converted into engagement. This finding positions teacher knowledge sharing not only as a professional behavior but also as a motivational resource that supports vigor, dedication, and absorption. Third, the significant moderating role of supervisor support shows that the motivational value of knowledge sharing depends partly on the level of support provided by school leaders and supervisors.

The findings also provide practical implications for schools. School leaders should strengthen leadership practices that combine moral guidance, relational care, and clear instructional support, because such practices can increase teachers’ willingness to exchange professional knowledge and remain engaged in their work. Schools should also establish structured knowledge-sharing mechanisms for calligraphy teachers, such as regular peer demonstration sessions, lesson study groups, teaching material exchange platforms, and cross-school professional learning communities. These mechanisms are especially important because calligraphy teaching relies heavily on tacit skills, aesthetic judgment, and demonstration-based pedagogy. In addition, supervisors should provide consistent feedback, emotional reassurance, and recognition of calligraphy teachers’ cultural and instructional contributions. When supervisor support is strong, the positive influence of knowledge sharing on work engagement becomes stronger, suggesting that professional collaboration should be accompanied by active supervisory encouragement.

Overall, the results indicate that improving calligraphy teachers’ work engagement requires coordinated attention to leadership behavior, collaborative knowledge structures, and supervisor support. Schools should not rely only on individual teacher motivation; they should create organizational conditions that help specialist teachers access resources, share expertise, and feel professionally valued.

## 7 Limitations and future research

Several limitations should be acknowledged. First, the study was conducted among in-service calligraphy teachers in Hebei Province, which may limit the generalizability of the findings to other regions, school levels, or subject areas. Future research should include teachers from multiple provinces and compare calligraphy teachers with teachers of other specialist and core subjects. Second, the cross-sectional design limits causal interpretation. Longitudinal or time-lagged designs could provide stronger evidence regarding how leadership, knowledge sharing, supervisor support, and work engagement develop over time. Third, the study relied on self-reported questionnaire data, which may introduce common method bias. Future studies may combine teacher surveys with supervisor ratings, classroom observations, or qualitative interviews. Fourth, the study focused on knowledge sharing and supervisor support as key resources, while other school-level factors such as professional identity, psychological safety, teaching workload, and institutional recognition may also influence work engagement. Future studies could examine these additional mechanisms to develop a more comprehensive understanding of specialist teacher engagement in culturally rooted educational contexts.

## 8 Conclusion

This study demonstrates that paternalistic leadership, knowledge sharing, and supervisor support play significant roles in shaping the work engagement of in-service calligraphy teachers. All proposed hypotheses were supported: paternalistic leadership significantly predicted knowledge sharing and work engagement; knowledge sharing significantly predicted work engagement and mediated the relationship between paternalistic leadership and work engagement; and supervisor support significantly strengthened the relationship between knowledge sharing and work engagement. These findings indicate that teacher engagement in calligraphy education is shaped by a combination of relational leadership, collaborative professional exchange, and supportive supervisory conditions. By extending the JD-R model to a culturally specific educational context, the study contributes to understanding how school-based resources support the motivation and professional sustainability of specialist teachers. The results suggest that improving calligraphy education requires organizational structures that enhance professional interaction, strengthen supervisor support, and compensate for gaps in formal training and institutional recognition.

## Data Availability

The original contributions presented in the study are included in the article/supplementary material, further inquiries can be directed to the corresponding author/s.
